# Citrullinated histone H3 and elevated polyamine metabolism gene expression in peripheral blood polymorphonuclear leukocytes as biomarkers for poor immunotherapy response in cancer patients

**DOI:** 10.1186/s43556-026-00644-9

**Published:** 2026-10-23

**Authors:** Amy Anzovino, Stephanie Kjelstrom, Kathryn Fleck, Margaretha Wallon, Eric T. Alexander, Susan K. Gilmour

**Affiliations:** Lankenau Institute for Medical Research, 100 Lancaster Avenue, Wynnewood, PA 19096 USA

**Keywords:** Polyamines, Glutamine, Neutrophil extracellular traps, Blood biomarkers, Cancer immunotherapy

## Abstract

There is a critical need to identify validated biomarkers that can predict cancer patient outcomes. This study evaluated biomarkers of neutrophil extracellular traps (NETs) and polyamine gene expression in peripheral blood that may correlate with greater risk for poor response to immunotherapy. Peripheral blood collected from cancer patients being treated with chemo/immunotherapy and from healthy volunteers was analyzed for levels of citrullinated histone H3 (Cit H3, a NETosis marker), inflammatory cytokines, and circulating tissue factor positive microparticles (TF^+^MPs). Elevated levels of plasma TF^+^MPs, Cit H3, IL-8, and IL-6 each significantly correlated with poor response to immunotherapy. Since in vitro stimulation of both human and mouse neutrophils to form NETs was blocked with addition of an inhibitor of polyamine uptake (Trimer44NMe), RNA expression of polyamine/glutamine metabolism genes was evaluated in both peripheral blood mononuclear cells (PBMCs) and polymorphonuclear leukocytes (PMN). Elevated expression of glutamine-associated genes in PBMCs and decreased expression of polyamine-associated genes in PMN correlated with favorable patient response to immunotherapy. Although not reaching significant levels, a high overall polyamine score in peripheral PMNs as well as increased plasma Cit H3 levels correlated well with poor response to immunotherapies in cancer patients. In a mouse model of triple negative breast cancer, polyamine blocking therapy inhibited not only primary tumor growth and lung metastases but also decreased intratumor NETs. Results from this study suggest a mechanistic link between NETosis and polyamine gene expression in PMN that warrant further investigation as peripheral blood biomarkers for immunotherapy response.

**Supplementary Information:**

The online version contains supplementary material available at https://doi.org/10.1186/s43556-026-00644-9.

## Introduction

While immunotherapy has resulted in durable remission for some cancer patients, many patients still do not benefit from immune checkpoint inhibitors (ICI) or suffer significant life-threating immunotoxicity [1]. There is an urgent need to identify new biomarkers to predict the response to ICI, allowing physicians to select the best anti-cancer agents for each patient. Multiple studies support a central role for neutrophil recruitment and activation in cancer therapy resistance and metastasis [2–6]. Neutrophils can undergo a unique form of cell death resulting in the formation of neutrophil extracellular traps (NETs) in which strands of DNA-histone complexes and antimicrobial peptides and proteases are released into the extracellular space to trap and kill microorganisms. NETs are often abundant in tumor tissue (in the absence of infection) and have been correlated with tumor progression and metastasis in many types of cancer [7–12]. It has been proposed that NETosis may be an underlying mechanism of cancer-mediated thrombosis that, in turn, also drives tumor progression [9, 13, 14]. Moreover, the interaction of NETs with tumor cells is thought to induce resistance to chemotherapy/immunotherapy [6], and NETosis markers in peripheral blood have been associated with chemotherapy resistance and poor survival in cancer patients [15–17].

Polyamines, small molecules that can potentially stabilize NETs [18], are dramatically elevated in most tumors, suggesting a link between polyamines, NETs, and cancer progression. Polyamines can also activate peptidylarginine deiminase 4 (PAD4) resulting in citrullination of histones and triggering NETosis [19]. Due to their polycationic properties, polyamines (putrescine, spermidine, and spermine) interact with DNA, RNA and proteins, thus strongly influencing chromatin remodeling, ion channel regulation, cellular proliferation and survival, and immune cell modulation [20, 21]. Studies using murine tumor models demonstrate that increased polyamine biosynthesis in the tumor microenvironment (TME) is associated with increased numbers and survival of immunosuppressive myeloid derived suppressor cells (MDSCs), M2-macrophages, and regulatory T-cells (Tregs) as well as a decrease in anti-tumor cytotoxic T-cell activity [20–24]. Polyamines modulate leukocyte polarization within the TME to promote a pro-tumorigenic M2 phenotype [24–27]. Altogether, these suggest a possible link between elevated tumor polyamine metabolism, NETosis, and creation of an immunosuppressive TME that supports cancer progression and metastasis.

For many years the polyamine metabolic pathway has been targeted with various inhibitors to treat cancer patients [28]. The most successful and widely used inhibitor of polyamine biosynthesis is α-difluoromethylornithine (DFMO), an irreversible inhibitor of ornithine decarboxylase, the rate-limiting enzyme in polyamine biosynthesis [20, 29–31]. However, DFMO monotherapy has had only moderate clinical efficacy in treating cancers due to a compensatory increase in polyamine transport activity when polyamines are depleted [20, 32]. Promising preclinical studies to more effectively deplete tumor polyamine levels have highlighted the anti-tumor and immunomodulatory effects of a polyamine blocking therapy (PBT) that includes both DFMO and a polyamine transport inhibitor (PTI) [24, 33–40]. Because preclinical studies demonstrate that PBT can promote anti-tumor immune responses, inhibitors of polyamine metabolism are now moving into clinical trials [28, 41].

Recent studies have shown that high polyamine metabolism gene expression scores and/or high polyamine levels within tumor tissues of various cancer types are predictive for poor response to immunotherapy [42–47]. In addition to tumor biomarkers, there is a need to identify biomarkers in easily obtained blood samples that can distinguish cancer patients whose tumors will better respond to new polyamine targeted therapies. In this exploratory study, plasma, peripheral blood mononuclear cells (PBMCs), and polymorphonuclear leukocytes (PMN) from cancer patients and healthy volunteers were analyzed for biomarkers of NETosis, cytokine levels, and expression of polyamine metabolism gene expression. Our results suggest that plasma biomarkers of NETosis and polyamine-related gene expression in circulating PMN may be useful as prognostic indicators for immunotherapy response in cancer patients.

## Results

### Increased levels of NETosis-related factors and cytokines in cancer patients

**Fig. 1:**
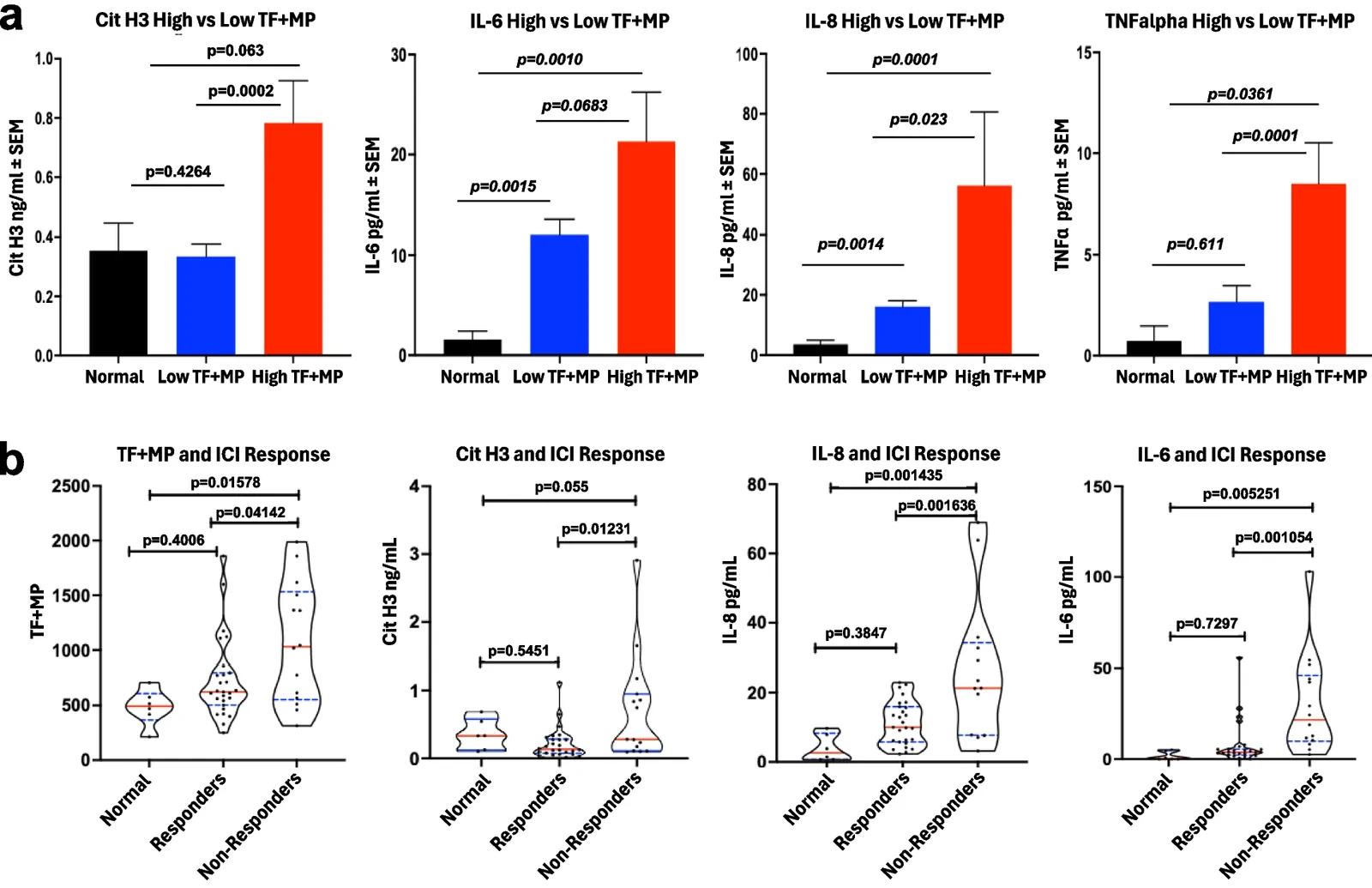
Elevated plasma Cit H3 levels, circulating TF^+^MPs, and IL-6 and IL-8 correlate with poor response to immunotherapy. **a** PPP samples from healthy volunteers (*n* = 6) and from patients with breast cancer, lung cancer, renal cell carcinoma, or mesothelioma (*n* = 60) were analyzed for total numbers of circulating TF^+^ MPs in 150 µl PPP and plasma levels of Cit H3 and cytokines. Circulating TF^+^ MPs were identified using flow cytometry. Cit H3 levels were analyzed by ELISA. Cytokines (IL-8, IL-6, and TNF-α) were analyzed using a cytokine bead array and flow cytometry. Mean ± SEM. **b** Correlation between blood levels of NETosis biomarkers and cytokines and response to immunotherapy in cancer patients. PPP samples from healthy volunteers and from cancer patients (*n* = 41) were analyzed for TF^+^ MPs, Cit H3, and cytokines IL-8 and IL-6. Total number of circulating TF^+^ MPs in 150 µl PPP were identified using flow cytometry. Cit H3 levels (ng/ml) in PPP were analyzed by ELISA. PPP levels of IL-8 and IL-6 (pg/ml) were quantified using a cytokine bead array and flow cytometry. Violin plots show the final blood levels measured for normal healthy volunteers (*n* = 6), for cancer patients responding favorably to therapy including ICIs (*n* = 27), and for cancer patients non-responsive to immunotherapy as defined by tumor progression and/or death (*n* = 14). Mean depicted by red line. In all 4 violin plots, there are no significant differences between Normal and Responder Cit H3, TF^+^ MPs, or cytokine levels

Blood samples were collected from 33 cancer patients being treated with immune checkpoint inhibitors as well as from healthy control subjects. Cancer types varied with 16 lung (including 15 non-small cell lung cancer), 9 triple negative breast cancer (TNBC), 4 renal, 2 melanoma, 1 mesothelioma, and 1 cholangiocarcinoma (Table 1). Platelet-poor plasma (PPP) was analyzed for levels of citrullinated histone H3 (Cit H3, a NETosis biomarker) and an array of inflammatory cytokines (including IL-12p70, TNFα, IL-10, IL-6, IL-8, and IL-1β). Since previous studies have demonstrated that NETosis can initiate coagulation via release of tissue factor positive microparticles (TF^+^ MPs) from tumor cells [48, 49], PPP was also analyzed for circulating TF^+^MPs (0.5 µm to 1.0 µm in size). Blood from healthy volunteers had low numbers of circulating TF^+^ MPs (< 1,200 in 150 µl PPP) and low plasma levels of Cit H3 (< 0.6 ng/mL) compared to that of some cancer patients. Moreover, there was a significant correlation between cancer patients with elevated numbers of plasma TF^+^MPs (≥ 1,200) and high levels of Cit H3 (Fig. 1a). We hypothesized that TF^+^ MPs stimulate not only coagulation but also the release of inflammatory cytokines such as IL-8, IL-6, and TNF-α, all of which, in turn, prime neutrophils to undergo NETosis releasing Cit H3 [4, 16, 50, 51]. Indeed, there was a strong correlation between high levels of circulating TF^+^MPs and inflammatory cytokines (IL-6, IL-8, and TNFα) (Fig. 1a). These results suggest that NETosis-stimulated coagulation and resulting increased numbers of circulating TF^+^MPs are not independent entities but may stimulate each other, thus driving tumor progression. Plasma Cit H3 levels and other NETosis markers have previously been reported to be associated with chemotherapy resistance and poor survival in cancer patients [15–17]. We confirmed that not only plasma Cit H3 but also high levels of circulating TF^+^MPs, IL-8, and IL-6, each significantly correlated with poor therapy response in this cohort of cancer patients (Fig. 1b). Although a sensitivity analysis stratified by cancer type is limited by low patient numbers, our results suggest that elevated plasma levels of the NETosis marker Cit H3, circulating TF^+^MPs, and IL-6 and IL-8 are markers for cancer patients with poor response to immunotherapies.

**Table 1:** Cohort characteristics of patient participants by therapy response. Responder status was defined using treatment response

Variable	Non-responders (*n* = 13)	Responders (*n* = 20)
Sex, n (%)		
Female	6 (46.2%)	10 (50.0%)
Male	7 (53.8%)	10 (50.0%)
Age, years		
Median (IQR)	71.0 (61.0–78.0)	64.5 (56.8–70.5)
Cancer type group, n (%)		
Lung cancer	6 (46.2%)	10 (50.0%)
Breast cancer (TNBC)	3 (23.1%)	6 (30.0%)
Other solid tumors	4 (30.8%)	4 (20.0%)
Treatment category, n (%)		
ICI monotherapy	4 (30.8%)	3 (15.0%)
ICI only, multiple ICI agents listed	0 (0.0%)	2 (10.0%)
ICI plus chemotherapy	9 (69.2%)	15 (75.0%)

Values are presented as n (%) the number of patients within each category (corresponding percentage of that category); median; and interquartile range (IQR). ICI includes immune checkpoint inhibitor therapy; *TNBC* triple-negative breast cancer

### Association between NET formation and polyamine accumulation

**Fig. 2:**
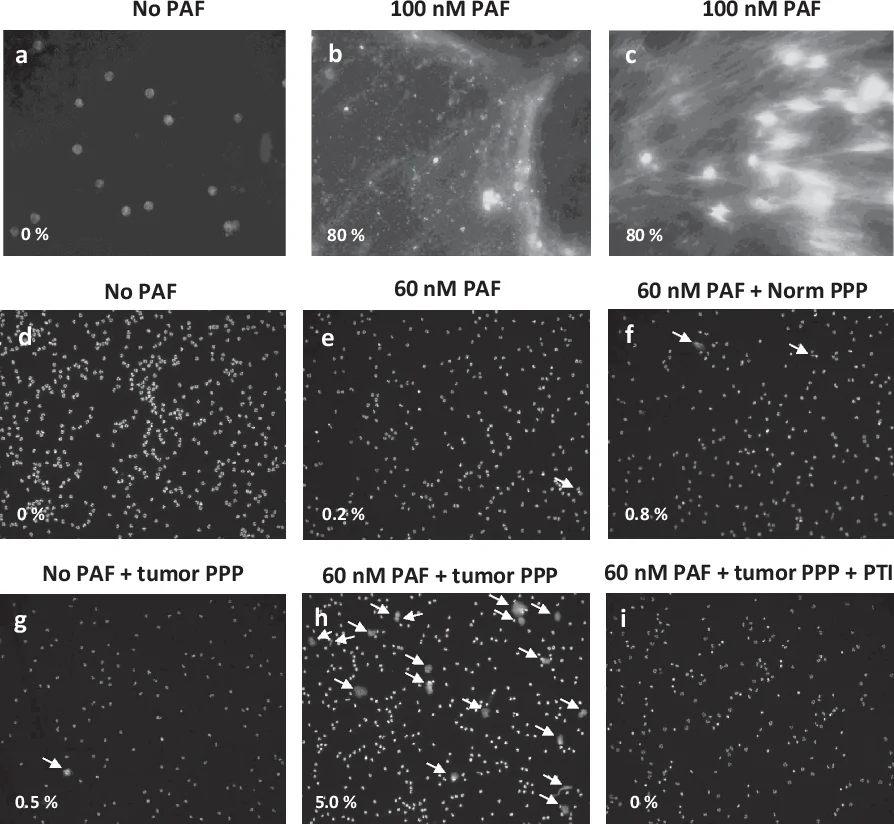
NETosis is blocked by an inhibitor of polyamine import. Neutrophils isolated from peripheral blood collected from healthy volunteers were stimulated to undergo NETosis following stimulation for 1 h with (**b**, **c**) 100 nM PAF; or (**e**, **f**, **h**, **i**) a subthreshold NETosis-inducing concentration of 60 nM PAF; or (**g**-**i**) 60 nM PAF ±7.5 µl PPP from a cancer patient (tumor PPP) ± 1 μM Trimer44NMe PTI (**i**); or (**f**) 60 nM PAF ±7.5 µl PPP from a healthy individual (Norm PPP). NETs (indicated with white arrows) were visualized (**a**, **b**) following immunofluorescent staining with an antibody against Cit H3 or (**c**-**i**) staining with PicoGreen. The percentage of neutrophils undergoing NETosis is indicated in the lower left of each panel

The presence of cytokines such as TGF-β and hypercoagulation within the TME can lead to polarization of neutrophils to a N2 pro-tumorigenic phenotype that can spontaneously undergo NETosis [10, 52]. We speculated that elevated levels of polyamines within tumors can also facilitate this metabolic shift in neutrophils that confers susceptibility to forming NETs. To investigate a possible link between NETosis and polyamines, neutrophils were isolated from the peripheral blood of healthy volunteers and stimulated to undergo NET formation with 100 nM platelet activated factor (PAF) (Fig. 2a-c). Since tumor-derived cytokines and other factors can stimulate NETosis [50], we investigated to what extent culturing neutrophils with PPP from cancer patients or healthy volunteers may predispose neutrophils to more readily undergo NETosis when stimulated with a subthreshold concentration of 60 nM PAF. As expected, exposure to cancer patient PPP, but not healthy PPP, greatly stimulated NETosis in PAF-treated neutrophils (Fig. 2c-g). We then investigated to what extent a polyamine targeted therapy would block NET formation by adding DFMO to inhibit polyamine biosynthesis or the Trimer44NMe PTI that blocks polyamine uptake into cells. Interestingly, DFMO addition did not block NETosis (data not shown), but treatment with the Trimer44NMe PTI completely inhibited in vitro NETosis (Fig. 2h).

**Fig. 3:**
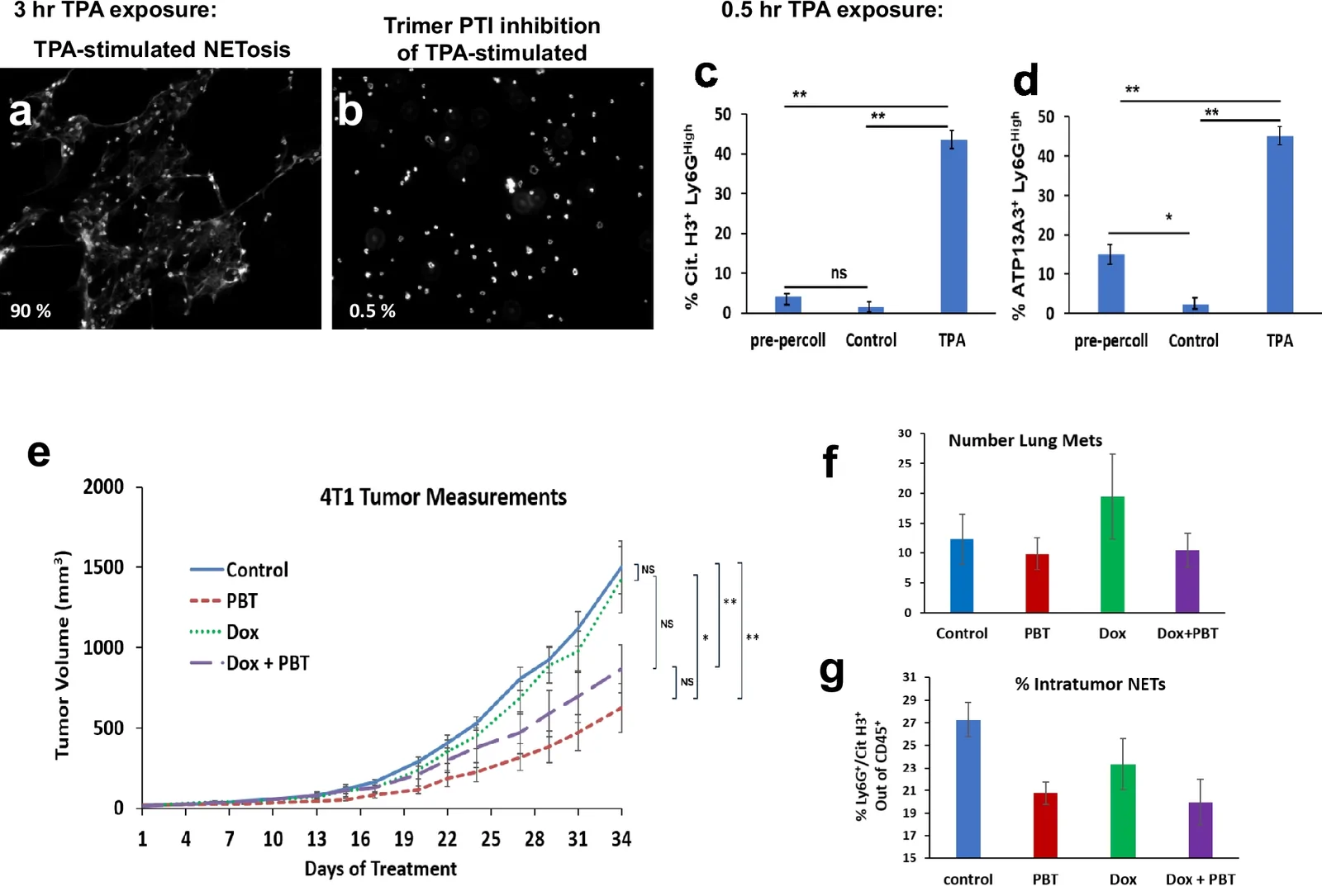
Early mechanistic link between NET formation and polyamine transport into neutrophils. PicoGreen fluorescent staining of murine bone marrow-derived neutrophils (more than 95–98% Ly6G^Hi^ by flow cytometry following Percoll gradient purification) stimulated for 3 h with (**a**) 100 nM TPA (resulting in 90% NETosis); and **b** 100 nM TPA + 1 uM Trimer44NMe PTI (resulting in 0.5% NETosis). **c** and **d** Flow cytometry analysis of bone marrow cells from C57Bl/6 mice before Percoll gradient purification and after separation with a Percoll gradient when purified neutrophils were plated in triplicate and stimulated for 0.5 h with control vehicle or 100 nM TPA. No NETs were detected following exposure to 100 nM TPA for only 0.5 h. Nonpermeabilized cells were analyzed by flow cytometry for surface expression of (**c**) Ly6G^High^ and Cit H3 and **d** Ly6G^high^ and ATP13A3. (*n* = 3; mean ± SD; ns: not significant, * *p* ≤ 0.05, ** *p* ≤ 0.005; using neutrophils purified from additional murine bone marrow cells, the experiment was repeated twice with similar results). **e** Female Balb/c mice were orthotopically injected with 1 × 10^4^ 4T1 tumor cells in the mammary fat pad. 10 days later, treatment was initiated with Dox (1.5 mg/kg, i.p., once every week) or PBT (0.5% DFMO in the drinking water + 3 mg/kg Trimer44NMe PTI, i.p., daily) or Dox + PBT. All mice were sacrificed following 34 days of treatment, and lungs and primary mammary tumors were examined for metastatic nodules and intratumor NETs (Ly6G^+^ Cit H3^+^ neutrophils), respectively. Tumor growth was measured with calipers from the time treatment was initiated until the mice were sacrificed. (*n* = 6 mice/treatment group; mean ± SEM; ns: not significant, * *p* ≤ 0.03, ** *p* ≤ 0.005). **f** Number of total lung metastases as visualized after injection of India ink following euthanasia. (*n* = lungs from 6 mice/treatment group; mean ± SEM; no significant difference between treatment groups). **g** Percentage of intratumor Ly6G^+^ Cit H3^+^ neutrophils out of all CD45.^+^ cells in the tumors was identified by flow cytometry. (*n* = tumors from 6 mice/treatment group; mean ± SEM; no significant difference between treatment groups)

In addition to human neutrophils, neutrophils isolated from mouse bone marrow were stimulated to form NETs by the addition of 12-O-tetradecanoylphorbol-13-acetate (TPA) (Fig. 3a). Once again, TPA-induced NET formation was completely inhibited with the Trimer44NMe PTI (Fig. 3b). ATP13A3 was recently identified as an important component of the polyamine transport system, and its transport function is inhibited with the Trimer44NMe PTI [34–36]. Flow cytometry analysis showed that cell surface expression of both ATP13A3 and Cit H3 was increased after only 30 min of TPA stimulation and before the extrusion of DNA-histone NETs (Fig. 3c). Altogether, these results suggest an early mechanistic link between NET formation and polyamine transport into neutrophils and implicate ATP13A3 as a putative target for NETosis-stimulated metastasis and therapy resistance.

### Polyamine blocking therapy alleviates doxorubicin-promoted metastasis and decreases 4T1 tumor NETs

In view of our in vitro NETosis results, we examined the effect of a polyamine targeted therapy on metastasis and NETosis in mice orthotopically implanted with syngeneic TNBC 4T1 cells. 4T1 tumor cells metastasize efficiently to the lungs [53], and this metastasis is stimulated by the formation and release of NETs [10]. Because TNBC patients are often treated with a standard chemotherapeutic agent such as doxorubicin (Dox), 4T1 tumor-bearing mice were treated with Dox (1.5 mg/kg, i.p., once every week) with and without PBT consisting of both the Trimer44NMe PTI and DFMO. Whereas 4T1 primary tumor growth was not inhibited using this low dose Dox, tumor growth was significantly inhibited with either PBT alone or Dox + PBT compared to that in vehicle-treated control mice (Fig. 3e).

Previous studies have reported that chemotherapy such as Dox activates TGFβ signaling in both mouse and human breast cancer, inducing epithelial-mesenchymal transition (EMT), invasion, and metastases. In addition, NETs have been shown to trap and activate latent TGF-β causing cancer cells to undergo EMT and metastasis [54, 55]. We confirmed that Dox treatment alone increases the number of lung metastases in mice bearing 4T1 tumors (Fig. 3f). Dox-induced lung metastases were inhibited by co-treatment with PBT, suggesting that polyamines may play a role in Dox-induced metastasis. (Fig. 3f). Analysis of intratumor neutrophils undergoing formation of NETs (Ly6G^+^ neutrophils expressing Cit H3) revealed that PBT alone and PBT + Dox inhibited NETosis compared to that in vehicle-treated mice (Fig. 3g). The similar pattern of PBT inhibition of Dox-induced metastasis and Dox-induced intratumor NETs, as well as the known association between NETosis and metastasis, suggests that polyamines contribute, at least in part, to tumor NETosis and resultant metastasis.

### Polyamine metabolic gene expression as biomarkers for predicted treatment response

**Fig. 4:**
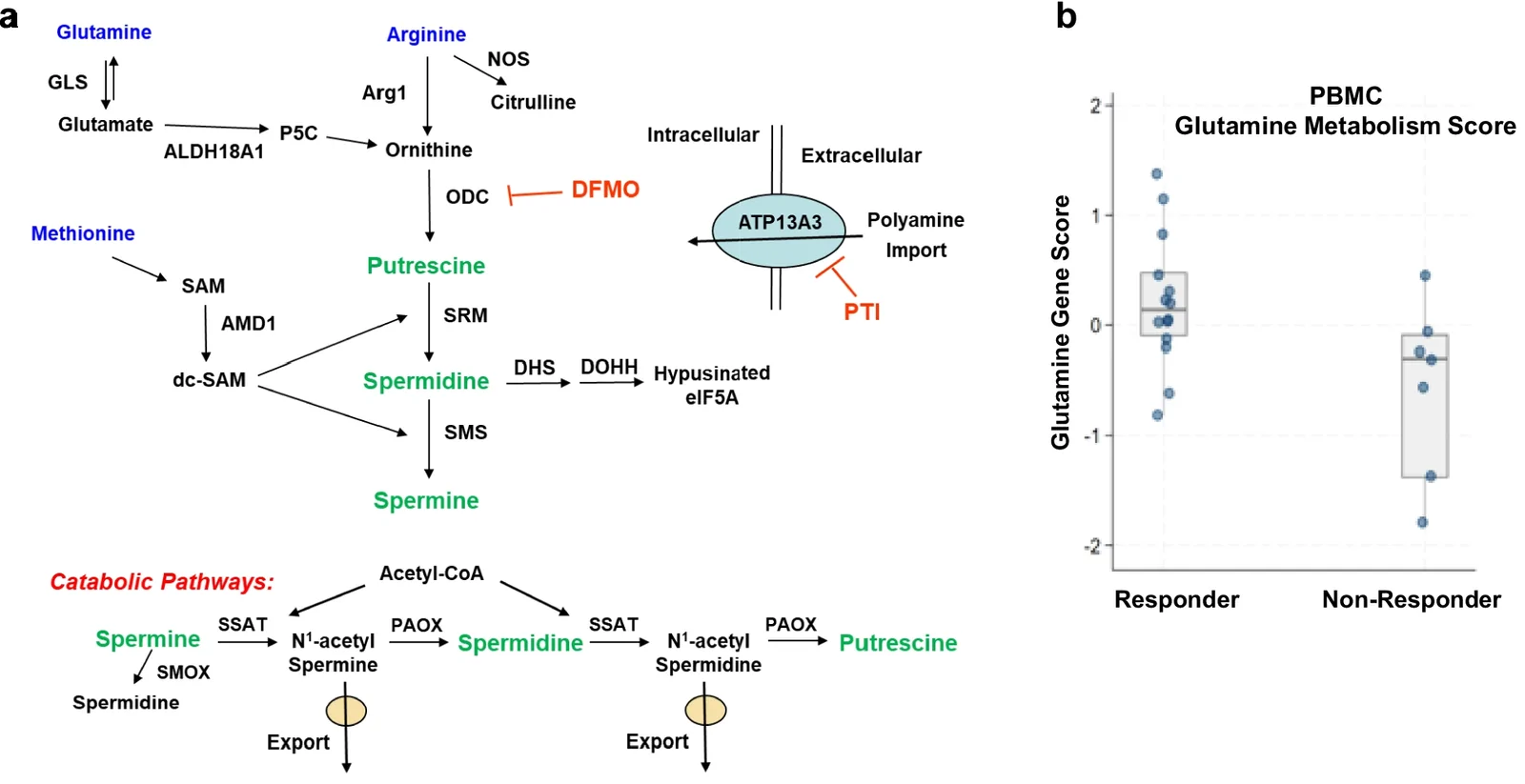
Elevated PBMC glutamine metabolism expression in therapy-responsive cancer patients. **a** Relationship between polyamine and glutamine metabolic pathways. Ornithine, the precursor of polyamines, can originate from glutamine or arginine catabolism. Spermidine and spermine are synthesized by the sequential additions of aminopropyl groups provided via decarboxylated S-adenosylmethionine (dc-SAM), which is derived from SAM and S-adenosylmethionine decarboxylase (AMD1). Putrescine, spermidine, and spermine can be imported into the cell via a polyamine transport system which includes ATP13A3. Following catabolic acetylation by SSAT, acetylated polyamines can be exported out of the cell or back-converted via PAOX catalytic oxidation to spermidine and putrescine. Spermidine is enzymatically converted to hypusine that post-translationally modifies (hypusinates) and activates only the translation factor eIF5A. ALDH18A1, aldehyde dehydrogenase 18A1; AMD1, adenosylmethionine decarboxylase 1; ARG, arginase; DFMO, α-difluoromethylornithine; DHS, deoxyhypusine synthase; DOHH, deoxyhypusine hydroxylase; GLS, glutaminase; NOS, nitric oxide synthase; OAT, ornithine aminotransferase; ODC, ornithine decarboxylase; P5C, pyrroline-5-carboxylate; PAOX, polyamine oxidase; PTI, polyamine transport inhibitor; SAM, S-adenosylmethionine; SMOX, spermine oxidase; SMS, spermine synthase; SRM, spermidine synthase; SSAT, spermidine/spermine N1-acetyltransferase 1. **b** The PBMC glutamine metabolism score was calculated as the mean of standardized log-transformed expression values for GLS, OAT, and ALDH18A1. The boxplot displays the glutamine pathway score medians by responder type and corresponds with the median values in Table 2. Gene expression values were aggregated at the patient level using median expression values prior to log transformation and standardization. Higher glutamine metabolism pathway scores were associated with treatment response

To investigate correlations between polyamine/glutamine metabolic gene expression in circulating leukocytes and tumor response to therapy, RNA was extracted from PBMCs and PMN isolated from 135 blood samples from the same pool of cancer patients and healthy volunteers as analyzed above (Table 1). Genes related to polyamine metabolism (Fig. 4a), in particular arginase-1 (ARG1), ornithine decarboxylase (ODC), the polyamine transporter ATP13A3, and spermine oxidase (SMOX), were elevated in both PBMCs and PMN of cancer patients compared to that in healthy control subjects (Supplementary Fig. S1 and Fig. S2). Although SMOX was elevated in a third of the cancer patient leukocytes, expression of other polyamine catabolic genes [spermidine/spermine N-acetyltransferase-1 (SAT1), and polyamine oxidase (PAOX)] was rarely increased compared to that in healthy control subjects. In both lymphocyte populations (PBMCs) and in PMN of cancer patients, elevated ODC expression correlated with elevated MYC expression (Supplementary Table S1). c-MYC is activated in many cancers and upregulates expression of multiple polyamine-related genes [56–60]. Elevated ODC and MYC expression were also accompanied by increased glutamine metabolism in PBMCs of these cancer patients. Glutamine catabolism is a hallmark of activation and metabolic reprogramming of T cells, and glutamine (not arginine) is the principal carbon source for polyamines in activated effector CD8^+^ T cells [61]. Large increases in ARG1 expression were often accompanied by an absence of increased expression in ODC or glutamine metabolism-related genes including glutaminase (GLS), aldehyde dehydrogenase (ALDH18A1), and ornithine aminotransferase (OAT). However, these data revealed limited evidence of strong individual gene-level associations with treatment response, supporting further evaluation using pathway-based and multivariable modeling approaches.

Polyamine pathway scores were constructed for PBMC and for PMN using standardized log-transformed expression values for ARG1, ODC, MYC, AMD1, ATP13A3, SMOX, SAT1, and PAOX (Table 2). In the PBMC analysis, the polyamine score did not differ between responders and non-responders (median 0.002 vs 0.007, *p* = 0.949), and logistic regression showed no significant association with treatment response (adjusted odds ratio [aOR] 1.0, (95% confidence interval [CI] 0.2, 4.7), *p* = 0.998). Similarly, in the PMN analysis, the polyamine score was numerically lower among responders compared with non-responders (−0.11 vs 0.05), although this difference was not statistically significant (*p* = 0.143). Logistic regression likewise showed a non-significant association between the PMN polyamine score and response (aOR 5.1, *p* = 0.098) (Table 2).

**Table 2:** Polyamine and glutamine pathway score medians and odds ratios associated with treatment response

Expression Score	n	Responder	n	Non-responder	p
Polyamine PBMC Score					
Median (IQR)	19	.002 (−0.3, 0.4)	11	.007 (−0.25, 0.4)	0.949
aOR (95% CI)	19	Reference	11	1.0 (0.2, 4.7)	0.998
Polyamine PMN Score					
Median (IQR)	14	−0.11 (−0.34, −0.04)	10	0.05 (−0.16, 0.95)	0.143
aOR (95% CI)	14	Reference	10	5.1 (0.8, 34.8)	0.098
Glutamine PBMC Score					
Median (IQR)	14	0.14 (−0.1, 0.5)	7	−0.3 (−1.4, −0.1)	0.037
aOR (95% CI)	14	Reference	7	0.1 (0.01, 1.1)	0.063
Glutamine PMN Score					
Median (IQR)	14	−0.07 (−0.6, 0.8)	7	−0.4 (−0.8, 0.2)	0.551
aOR (95% CI)	14	Reference	7	0.9 (0.3, 2.8)	0.851

*aOR* adjusted odds ratio, adjusted for age and cancer type (lung, breast, other)

*CI* confidence interval, *IQR* interquartile ranges

When analyzing glutamine metabolism gene expression (GLS, OAT, and ALDH18A1) in PBMCs, there was a significant association with treatment response (Table 2). Glutamine metabolism pathway scores were constructed using standardized log-transformed gene expression values and were higher in PBMCs from responder cancer patients compared to that in nonresponder patients, although after *p*-value adjustment, it was no longer significant (median 0.14 vs −0.3, *p* = 0.037). Figure 4b shows that glutamine-associated gene expression is more highly elevated in PBMCs from cancer patients that respond favorably to their immunotherapy compared to non-responsive cancer patients. When evaluated as a potential confounder, cancer type (grouped as lung, breast, and other solid tumors) was not significantly associated with treatment response or with the polyamine or glutamine pathway scores (all *p* > 0.05).

**Fig. 5:**
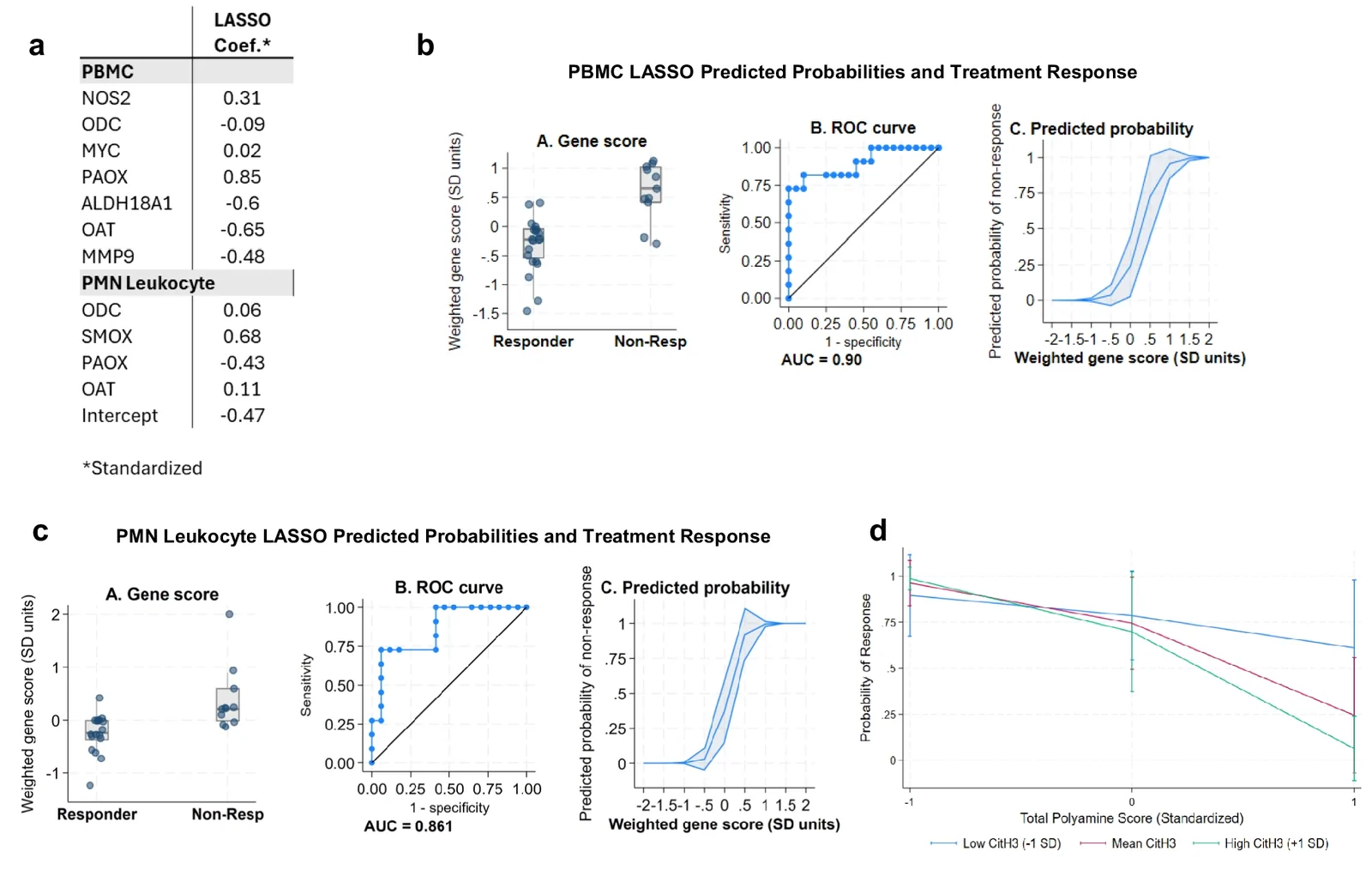
High overall PMN polyamine expression score and increased plasma Cit H3 levels correlate with poor response to immunotherapy. **a** Standardized LASSO coefficients were used to construct weighted gene scores for PBMC and for PMN leukocytes. **b** The PBMC weighted gene score was constructed using standardized penalized coefficients from the LASSO logistic regression model, which selected NOS2, ODC, PAOX, ALDH18A1, OAT, and MMP9 as the most predictive genes associated with treatment response. **c** The PMN leukocytes weighted gene score was constructed using standardized penalized coefficients from the LASSO logistic regression model, which selected ODC, SMOX, PAOX, and OAT as the most predictive genes associated with treatment response. **b** and **c** Panel A displays the distribution of weighted gene scores by responder status. Panel B shows the receiver operating characteristic (ROC) curve for the weighted gene score model (AUC = 0.861). Panel C illustrates predicted probabilities of non-response across weighted gene score values derived from logistic regression. **d** Graph showing that the relationship between the PMN polyamine score and Cit H3 differs by response status. Among responders, there was a moderately positive correlation between the polyamine score and Cit H3 (Spearman ρ = 0.40, 95% CI: − 0.02 to 0.77), whereas the association was weaker and more variable among non-responders (ρ = 0.17, 95% CI: − 0.46 to 0.71), although CIs were wide in both groups. In logistic regression models, there was evidence of an interaction between the polyamine score and Cit H3 (OR = 0.047, 95% CI: 0.002–1.30, *p* = 0.071), suggesting that the relationship between these variables and response is not constant. As levels of the polyamine score increased, the connection between Cit H3 and response became weaker

To identify the most predictive genes, least absolute shrinkage and selection operator (LASSO) logistic regression was performed using all measured gene expressions. For PBMC analysis, LASSO selected NOS2, ODC, PAOX, ALDH18A1, OAT, and MMP9. In the PMN analysis, the selected genes were ODC, SMOX, PAOX, and OAT. Standardized LASSO coefficients were used to construct weighted gene scores for PBMC and PMN (Fig. 5a). In logistic regression models, the weighted gene score was significantly associated with treatment response for both PBMCs (OR 2.6, 95% CI 0.87–4.3, *p* = 0.003) and PMNs (OR 3.3, 95% CI 0.5–6.2, *p* = 0.022). Model discrimination was high for both scores (Fig. 5b and c). We attempted to adjust the weighted gene scores with cancer type, and although the gene score remained significant, it was unstable; therefore, we did not report it here. The PBMC LASSO score demonstrated an area under the curve (AUC) of 89.6% (95% CI 75.7–100) with a cross-validated AUC of 85.5% (95% CI 48.4–100) (Fig. 5b). Similarly, the PMN LASSO score achieved an AUC of 86.1% (95% CI 72.0–100) with a cross-validated AUC of 82.5% (95% CI 68.9–100) (Fig. 5c). These findings suggest that the LASSO-derived weighted gene scores have good discriminatory ability for predicting treatment response.

Whereas the overall polyamine pathway score did not differ significantly between responders and non-responders, the LASSO-derived weighted gene score showed high predictive performance (AUC > 0.85). This difference arises because the overall pathway score weighs all genes equally, so informative genes are averaged together with uninformative or negatively associated ones, diluting the signal and biasing the group comparison toward the null. In contrast, LASSO selects and differentially weights only the most predictive genes, concentrating the discriminative signal and capturing multivariable information that a simple aggregate cannot. This contrast illustrates the advantage of penalized, data-driven biomarker selection over unweighted pathway aggregation and supports the LASSO-derived scores as candidate signatures for future validation.

Using the total polyamine score (fold increase of ARG, ODC, MYC, ATP13A3, SMOX, SAT1, PAOX, OAT in cancer patients compared to healthy subjects) for PMNs, we next investigated to what extent the relationship between the PMN polyamine score and Cit H3 may differ by response status. Figure 5d shows evidence that the association between Cit H3 and response was modified by the level of the PMN polyamine score. At low PMN polyamine score levels, the probability of good response to therapy remained uniformly high across Cit H3 values. In contrast, at higher PMN polyamine score levels, increasing Cit H3 was associated with a sharp decline in therapy response probability. This interaction did not reach conventional statistical significance (*p* = 0.07), likely due to limited sample size, but was consistent across multiple analytical approaches. These analyses suggest that responders have a more consistent pattern between the PMN polyamine score and Cit H3, whereas non-responders show more variation. In contrast, PBMC polyamine gene expression and Cit H3 were not significantly correlated within responders or nonresponders. Moreover, unlike Cit H3, neither total cytokine levels nor numbers of circulating TF^+^MPs show a response-dependent association with PMN polyamine gene expression in this cohort of cancer patients. Although not reaching significant levels, these analyses suggest that a high overall polyamine score in peripheral PMNs as well as increased plasma Cit H3 levels correlate well with poor response to immunotherapies in cancer patients.

## Discussion

High levels of polyamines and elevated expression of polyamine metabolism genes in tumor tissues have been found to be positively correlated with poor prognosis and unfavorable response to immunotherapy in a variety of cancers [42–47]. In addition, elevated levels of polyamine metabolites in the blood or urine have been proposed as early tumor biomarkers for ovarian, breast, and colon cancer [44, 62, 63]. However, this is the first study to explore the correlation between polyamine metabolic gene expression in circulating leukocytes and therapeutic response to cancer immunotherapy. Peripheral blood biomarkers offer several advantages including the availability of blood samples and the ability to measure biomarkers multiple times over the course of treatment compared to other methods such as tumor biopsies or tissue sampling. Importantly, this study provides compelling data that warrant future clinical trials to evaluate how biomarkers of NETosis and polyamine metabolism correlate with tumor progression and immunotherapy response.

Multiple studies show that polyamines support the growth and function of immunosuppressive macrophages, MDSCs, and neutrophils that inhibit the activity of T cells and dendritic cells and modulate the immune landscape by activating fibroblasts and endothelial cells [21, 26, 41, 64, 65]. In part this is due to polyamine-regulation of epigenetic enzymes that alters gene expression leading to metabolic reprogramming [66, 67]. In addition, polyamine-dependent hypusinated eIF5A induces mitochondrial oxidative phosphorylation that stimulates M2 polarization in myeloid cells including macrophages and neutrophils [68]. Our analyses show that elevated expression of LASSO-selected polyamine metabolic genes (ODC, SMOX, PAOX, and OAT) in PMN correlates with poor response to immunotherapy.

In contrast, polyamine metabolism has a direct effect on T-cell phenotype and the ability of T-cells to kill tumor cells [61, 69]. In this study, elevated expression of glutamine-associated genes (GLS, OAT, and ALDH18A1) in PBMCs, but not PMNs, significantly correlates with good response to immunotherapy in cancer patients. This is consistent with a report showing that the primary carbon source for polyamines in activated effector CD8^+^ T cells is via glutamine catabolism [61].

**Fig. 6:**
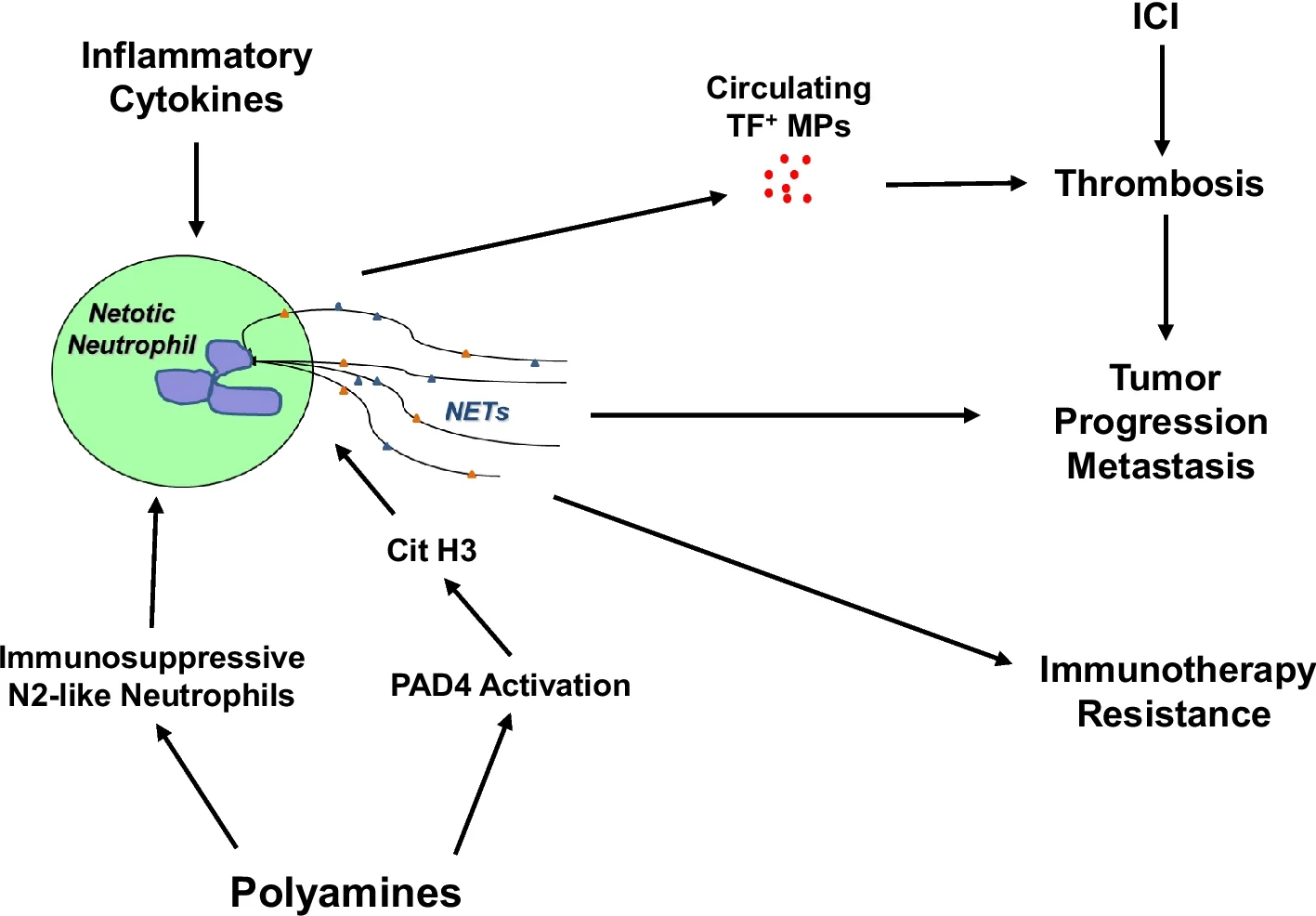
Diagram illustrating possible relationships between polyamines, inflammatory cytokines, NETosis, and immunotherapy resistance and tumor progression/metastasis. Polyamines can polarize neutrophils to a N2-like phenotype, making them more susceptible to undergoing NETosis. Polyamines can also activate PAD4 leading to citrullination of histones. NETs promote metastasis by a variety of mechanisms and initiate coagulation via release of TF, triggering thrombosis that, in turn, drives tumor progression and metastasis

Considering that the majority of PMN in peripheral blood are neutrophils, we were interested if PMN polyamine gene expression correlates with plasma levels of the NETosis biomarker, Cit H3 and treatment response. We found a strong association between elevated expression of PMN polyamine metabolic genes and plasma Cit H3 levels that correlates with poor response to immunotherapy. The early cell surface expression of both ATP13A3 and Cit H3 in NETosis-stimulated neutrophils as well as the inhibition of NETosis by the Trimer44NMe PTI suggests that polyamine import plays a role in the formation of NETs. This early increased expression of ATP13A3 is significant since the polyamine transport protein ATP13A3 translocates from the cytoplasm to the cell membrane when cellular polyamine import is stimulated [35]. The common mechanistic associations between polyamines, NETosis, and tumor progression (Fig. 6) aligns with the diagnostic potential of PMN expression of polyamine metabolic genes, plasma Cit H3 levels, and poor response to immunotherapy in specific cancers.

Since ICI-resistant murine tumors have previously been shown to be resensitized to ICI therapy with PBT co-treatment [38], future studies are warranted to investigate to what extent PTI-inhibition of NETosis plays a role in the PBT-reversal of ICI tumor resistance and enhanced anti-tumor efficacy. Studies are underway to identify therapeutic agents targeting NETs or inhibiting their formation to enhance response to immunotherapies in the clinic [10, 70]. However, understanding the underlying mechanisms by which NETs influence tumor immunotherapy resistance and also achieving a safe and effective anti-tumor therapy remains a challenge. Since NETosis can stimulate metastasis and tumor progression, our finding that both Dox-induced metastasis and NETosis are inhibited by PBT suggests that PBT may be useful in reducing metastasis and tumor progression in cancer patients with elevated blood levels of the NETosis marker Cit H3 and polyamine gene expression in circulating PMNs.

Some limitations of this study are that the patient cohort included multiple cancer types treated with a range of ICI regimens and that baseline response rates to immunotherapy differ across malignancies. Because several tumor-specific subgroups were very small, cancer types were collapsed into broader categories (lung, breast, and other solid tumors), and formal analyses stratified by individual cancer type were not statistically reliable. Residual confounding by tumor type therefore cannot be fully excluded, and our findings should be considered exploratory and hypothesis-generating, requiring validation in larger disease-specific cohorts. Similarly, the heterogeneity of ICI-based regimens and the small number of outcome events precludes adjustment for treatment line, combination therapy, or timing, which represent additional potential sources of residual confounding. Future larger scale studies will need to address these limitations and further investigate the role in which dysregulated polyamine metabolism may make neutrophils more susceptible to forming NETs.

In conclusion, this study suggests a mechanistic link between NETosis and polyamine gene expression in leukocyte subpopulations that may serve as peripheral blood biomarkers to predict cancer patient therapy response. Use of these NETosis and polyamine metabolic biomarkers in peripheral blood may accelerate approval of new indications for novel or existing pharmaceutical drugs.

## Methods

### Blood collection from cancer patients and healthy volunteers

Ethical approval was obtained and maintained throughout the study period from the Lankenau Institutional Review Board (Reference ID: F/N-R12-3146L). Written informed consent was obtained from all participants prior to enrollment. Participants were informed that all collected data and test results would remain confidential and used solely for research purposes. Inclusion criteria were: (1) written informed consent; (2) histologically proven carcinoma, stage I-IV disease; (3) RECIST (version 1.1) measurable disease if stage IV; (4) met the criteria for PD-1/PD-L1; (5) planned treatment with appropriate chemo/immunotherapy. Exclusion criteria included prior chemo/immuno-therapy or radiation therapy, with the exception of palliative radiation therapy for sites of pain terminated at least two weeks prior to start of chemotherapy. Personal identifiers were replaced with study ID numbers on all biological samples.

Each participant donated a tube of blood (12 mg K_2_EDTA) prior to each cycle of therapy. All blood samples were collected prior to administration of antiemetics and therapeutic agents, including immunotherapies (approximately every 2–3 weeks). PPP was prepared from anti-coagulated blood (double centrifugation at 3000 × g for 15 min) and frozen at −80ºC for later analyses. In addition, PBMCs and PMN were isolated from whole blood using PolymorphPrep gradients following the manufacturer’s protocol (PROGEN, Heidelburg, Germany). Patients were stratified to responders and non-responders based on RECIST criteria where partial/complete response patients were considered responders, and progressive disease patients were considered non-responders.

### Analysis of TF^+^MP

For flow-cytometric analysis, microparticles were isolated from 150 µl of PPP by sequential centrifugation [71], diluted with Annexin V binding buffer (eBioscience), and then stained with APC-conjugated Annexin V (eBioscience) and PE-conjugated anti-TF antibody (RD systems) [72]. Samples were analyzed using a BD FACSCanto II and FACSDiva software. Microparticles were identified as annexin V positive events between 0.5 µm and 1.0 µm. Microparticles smaller than 0.5 µm were not included in the analysis due to difficulties distinguishing from background noise. Gate size was adjusted using fluorescent microbeads measuring 0.5, 1.0 and 3.0 µm (Molecular Probes). TF^+^MP thresholds were set from annexin V binding positive microparticles.

### Cytokine and Cit H3 analyses

PPP samples were analyzed for interleukin-8 (IL-8), interleukin-1β (IL-1β), interleukin-6 (IL-6), interleukin-10 (IL-10), tumor necrosis factor (TNF), and interleukin-12p70 (IL-12p70) protein levels using the Human Inflammatory Cytokines Cytometric Bead Array reagents (BD Biosciences, San Jose, CA) and flow cytometry as per the manufacturer’s protocol. The same PPP samples were also quantified for levels of Cit H3 using an ELISA kit (Cayman Chemical, cat. # 501,620) [16]. We defined high TF^+^MP as ≥ 1,200 in 150 µl PPP, and high Cit H3 as over 0.6 ng/mL, as these values were the highest “outside” values that we encountered in more than 20 non-cancer volunteers who donated blood. Both TF^+^MPs and Cit H3 can be transiently elevated with infections in volunteers with no cancer. With this defined cutoff, there was no significant difference between cancer patients with low TF^+^MPs or low Cit H3 and that found in control healthy volunteers.

### Animals

Six to eight-week-old immunocompetent female C57Bl/6 and Balb/C mice were purchased from Charles Rivers/NCI. Animals were housed 4 mice per cage in a room with controlled temperature and humidity under a 12 h light/12 h dark cycle. Protocol A91-550 for the use of mice in these studies was reviewed and approved by the Institutional Animal Care and Use Committee of the Lankenau Institute for Medical Research in accordance with the current US Department of Agriculture, Department of Health and Human Service regulations and standards.

### Cell culture

4T1 murine mammary carcinoma cells were obtained from the American Type Culture Collection (Rockville, MD). 4T1 cells were cultured in DMEM supplemented with 10% fetal bovine serum and 1 × Penicillin/Streptomycin (Cellgro). Cell authentication and IMPACT tests were performed before use of these cells in animal experiments. The cells were thawed from early passage cells, cultured for less than 2 months, and morphologic features checked regularly to avoid cross-contamination or misuse.

### Drugs

Working solutions of DFMO (donated by Dr. Patrick Woster, University of South Carolina) were prepared in sterile water. The Trimer44NMe PTI was synthesized as previously described [37] and supplied by Dr. Otto Phanstiel (University of Central Florida), and 1 mM stock solutions were prepared in sterile PBS and stored at −20 °C. Doxorubicin Hydrochloride solution for injection (2 mg/ml) was purchased from Pfizer Pharmaceuticals.

### In Vivo 4T1 tumor model

Eight-week old female Balb/c mice were orthotopically injected with 1 × 10^4^ 4T1 cells in 50 µl PBS in the mammary fat pad. When tumors were palpable (average tumor volume of 30–50 mm^3^), mice were randomized into treatment groups with closely size-matched tumors (*n* = 6 mice/treatment group), and treatment initiated with PBT (0.5% (w/v) DFMO in the drinking water and 3 mg/kg Trimer44NMe PTI in PBS, daily by intraperitoneal [i.p.] injection) and/or Dox (1.5 mg/kg, i.p., weekly). Tumor growth was assessed morphometrically using calipers twice a week, and tumor volumes were calculated using the formula V (mm^3^) = π/6 × A x B^2^ (A is the larger diameter and B is the smaller diameter) [73]. Following 34 days of treatment, all mice were euthanized, and tumors and lungs removed for analyses of immune infiltrates and metastases. Lung metastases were visualized after injection of India ink following euthanasia, and metastatic nodules counted by two independent people blinded to treatment group.

### Analysis of immune cells

Tumor tissues were digested in a 0.3% collagenase/0.1% hyaluronidase solution, pressed through a nylon mesh filter to obtain a single cell suspension, incubated in red cell lysis buffer (0.17 M Tris–HCl, 0.16 M NH_4_Cl) for 3 min, spun down, and resuspended in FACS buffer (PBS +1.5% FBS). Equal numbers of viable cells were stained with a viability dye and combinations of the following antibodies: Ly6G-APC (eBioscience, 17-5931-81), Ly6C-PerCP-Cy5.5 (eBioscience, 45-5932-82), CD45-PE-Cy5 (eBioscience, 15-0451-83), Cit H3 (Abcam, ab5103), and ATP13A3-PE (Sigma, HPA029471). Flow cytometric data were acquired using a BD FACSCanto II cytometer and analyzed with FACSDiva software (BD Biosciences).

### Neutrophil isolation

Murine neutrophils were isolated from the bone marrow of 8-week-old C57BL/6 mice [74]. Bone marrow was flushed from both femurs with 1 × Hanks’ Balanced Salt Solution (HBSS) using a syringe with a 26G needle. After washing the bone marrow cells with 1 × HBSS, cells were resuspended in hypotonic 0.2% NaCl lysis buffer for 20 s on ice. Following two washes with 1 × HBSS, two mL of bone marrow cells were layered on top of a 52%–64%–72% Percoll (#17-0891-02, GE Healthcare) gradient followed by centrifugation at 1,500 × g for 30 min at 20 °C. Neutrophils were removed from the 64%/72% interface, washed in HBSS, and resuspended in serum-free RPMI before use in the NET formation assays described below. Isolated neutrophils had the typical segmented nuclei of mature neutrophils by microscopy and were 95–98% Ly6G^HI^ by flow cytometry.

Human neutrophils were purified from peripheral blood of healthy donors using PolymorphPrep gradients. The lower leukocyte band was collected, and red blood cells were lysed with ammonium-chloride-potassium lysis buffer for 3 min on ice. Isolated neutrophils were 96% CD16^+^ by flow cytometry.

### NET formation assay

Briefly, 200,000 neutrophils were seeded per well on 8-well CC2 glass chamberslides (Nunc Lab-Tek II CC2™ chamber slide system, cat.# 12-565-1, Thermo Scientific) in RPMI supplemented with 0.5% FBS for 30 min for adherence followed by addition of NETosis stimulators (vehicle, TPA, or PAF). NET inhibitors such as the Trimer44NMe PTI were added to the medium 30 min before neutrophil activation with either TPA or PAF. NETosis was assessed 0.5 to 4 h after initiating NET induction by fixing cells in chamberslides using 4% p-formaldehyde for 10 min at room temperature followed by 3 washes in PBS and then staining with the DNA-binding dye PicoGreen for 5 min. For immunofluorescence staining, slides were incubated in a blocking solution followed by anti-Cit H3 antibody in blocking buffer for 2 h and then stained with fluorochrome-conjugated secondary antibody (1:400, Thermo Fisher Scientific) for 1 h. Slides were covered with mounting medium (P36961, Thermo Fisher Scientific). Images were collected at 40 × magnification using a Zeiss Phase epifluorescence microscope and processed with Axioplan image analysis software. NETosis was determined as the percentage of the field of view positive for the merged signal of Cit H3 and PicoGreen.

### RNA isolation and quantitative PCR analysis

Following separation on PolymorphPrep gradients, PBMCs and PMN were analyzed by flow cytometry and were determined to be 94–98% CD3^+^ lymphocytes and CD16^+^ neutrophils, respectively. Isolated PBMCs and PMN were then analyzed by quantitative PCR (qPCR) to determine the relative mRNA levels of several polyamine/glutamine metabolism-related genes (*arg1, nos2, odc1, myc, amd1, atp13a3, smox, sat1, paox, gls, oat,* and *aldh18a1*) in cancer patients receiving ICI therapy compared to that in healthy volunteers. Total RNA was isolated from PBMCs and PMNs using TRIzol reagent (Invitrogen, 15,596,028) with the Direct-zol RNA MiniPrep kit (Genesee Scientific), and 1 µg of RNA was converted to cDNA using the GoScript Reverse Transcription System (Promega, A5000) as recommended by the suppliers. Primers (listed in Supplementary Table S2) were purchased from IDT and used in conjunction with SYBR™ Green JumpStart Taq ReadyMix (Sigma-Aldrich, S44388-500RXN) on a QuantStudio 3 (Applied Biosystems). Relative differences were calculated normalized to values for beta-2-microglobulin (B2M) by the comparative threshold cycle method. Previous studies have shown that B2M gene expression remains unchanged in immune cells including PBMCs and regulatory T cells (Tregs) following activation [75, 76]. We confirmed stable values for B2M expression in leukocytes isolated both from healthy and from participating cancer patients regardless of cancer type or treatment.

### Statistical analyses

Cytokine, Cit H3, and TF^+^MP analyses using PPP were performed at least in duplicate, and data were compiled from three to five separate samples. Analyses were done using Kruskal–Wallis test or a Dunn’s test for the multiple comparisons test or a 1-way ANOVA with a Tukey test to assess the statistical significance among groups using GraphPad Prism software (v8; GraphPad Software, Inc., La Jolla, CA, USA). Statistical significance was defined as p ≤ 0.05. In vivo studies were carried out using multiple animals (*n* = 6 mice per treatment group based on previous repeated murine tumor experiments).

Gene expression measurements were first aggregated at the patient level by calculating the median expression value for isolated PBMCs and PMNs for each patient. To reduce right skew, the influence of extreme values, and ensure gene expressions were on a common scale, gene expression values were log-transformed and standardized prior to analysis. Polyamine pathway scores were constructed by calculating the mean of the log-transformed standardized expression values for *ARG1, ODC, MYC, ATP13A3, SMOX, SAT1,* and *PAOX*. Glutamine metabolism pathway scores were similarly derived using *GLS, OAT,* and *ALDH18A1* expression values. Because of the limited sample size, continuous variables were summarized using medians and IQRs and compared between responders and non-responders using Wilcoxon rank-sum tests. As a sensitivity analysis, multivariable logistic regression models were used to evaluate associations between pathway scores and treatment response while adjusting for cancer type and age and reported as aORs with 95% CIs.

Because the number of candidate biomarkers was large relative to the sample size and the predictors were highly correlated, we used LASSO logistic regression, which reduces overfitting and performs automatic variable selection to identify a parsimonious set of predictors of treatment response. LASSO applies an L1 penalty that shrinks weak coefficients to zero, with the penalty parameter (lambda) selected by cross validation with ten folds to optimize predictive performance. These analyses were exploratory for candidate signatures for future validation. Logistic regression models were then fit using the weighted gene scores as predictors of treatment response. Weighted gene scores were visualized using boxplots stratified by responder status, receiver operating characteristic (ROC) curve plots, and predicted probability plots derived from logistic regression models. Model discrimination was evaluated using ROC curve analysis and AUC estimates with 95% CIs. Internal validation was performed using cross-validated AUC analyses. Statistical significance was adjusted for multiple comparisons using a Benjamin-Hochberg method (FDR < 0.05), and *p*-values < 0.024 were considered statistically significant. Statistical analyses were conducted in Stata (Statacorp, LLC, College Station, TX).

## Supplementary Information

Supplementary Material 1

## Data Availability

Data supporting this study’s findings are available from the corresponding author upon reasonable request. No new algorithms were developed for this study.

## Abbreviations

ALDH18A1: Aldehyde dehydrogenase 18A1
AMD1: Adenosylmethionine decarboxylase 1
aOR: Adjusted odds ratio
ARG1: Arginase-1
AUC: Area under the curve
Cit H3: Citrullinated histone H3
CI: Confidence interval
DFMO: α-Difluoromethylornithine
Dox: Doxorubicin
ECM: Extracellular matrix
EMT: Epithelial-mesenchymal transition
G-CSF: Granulocyte colony-stimulating factor
GLS: Glutaminase
GRO-α and GRO-β: Growth regulated-alpha and beta
HBSS: Hanks’ Balanced Salt Solution
HMGB1: High mobility group box-1
ICI: Immune checkpoint inhibitor
IL-6: Interleukin-6
IL-8: Interleukin-8
IQR: Interquartile range
LASSO: Least absolute shrinkage and selection operator
MDSCs: Myeloid derived suppressor cells
MMP-9: Matrix metalloproteinase-9
NET: Neutrophil extracellular trap
NOS2: Nitric oxide synthase-2
OAT: Ornithine aminotransferase
ODC: Ornithine decarboxylase
OR: Odds ratio
PAD4: Peptidylarginine deiminase 4
PAF: Platelet activating factor
PAOX: Polyamine oxidase
PBMC: Peripheral blood mononuclear cell
PBT: Polyamine blocking therapy
PMN: Polymorphonuclear leukocytes
PPP: Platelet poor plasma
PTI: Polyamine transport inhibitor
qPCR: Quantitative PCR
ROC: Receiver operating characteristic
SAT1: Spermidine/spermine N-acetyltransferase-1
SMOX: Spermine oxidase
TF: Tissue factor
TF^+^MP: Tissue factor positive microparticle
TME: Tumor microenvironment
TNBC: Triple negative breast cancer
TNFα: Tumor necrosis factor α
TPA: 12-O-tetradecanoylphorbol-13-acetate
Tregs: Regulatory T- cells
