# Supplementary material for: Citrullinated histone H3 and elevated polyamine metabolism gene expression in peripheral blood polymorphonuclear leukocytes as biomarkers for poor immunotherapy response in cancer patients
Source: Mol Biomed. 2026 Oct 23;7:223. doi: 10.1186/s43556-026-00644-9 (PMC13694611; doi:10.1186/s43556-026-00644-9)
Supplement: Supplementary file 1 [file 43556_2026_644_MOESM1_ESM.pdf]

Citrullinated Histone H3 and Elevated Polyamine Metabolism Gene Expression in Peripheral Blood Polymorphonuclear Leukocytes as Biomarkers for Poor Immunotherapy Response in Cancer Patients

Amy Anzovino[1], Stephanie Kjelstrom[1], Kathryn Fleck[1], Margaretha Wallon[1], Eric T. Alexander[1], and Susan K. Gilmour[1]

[1]Lankenau Institute for Medical Research, 100 Lancaster Avenue, Wynnewood, PA 19096

Supplementary Information:

Supplementary Tables

Supplementary Figures

Supplementary Table Legends

Supplementary Figure Legends

Supplementary Table S1

Patient ID number	Cancer Type	Treatment	ARG1	ODC	MYC	GLS	ALDH18A1	OAT
174-79	NSCLS	(Keytruda/Alimta)		4.5	2.5	12.1	3.7	
324-32	SCLS	(Tecentriq)		3.6	4.1		4.9	
326-17	Kidney	(Keytruda)	748					
382-47	Renal Cell Carcinoma	(Keytruda)		4.5	3.9	24.7	4.8	
394-10	NSCLC	(Keytruda)	7	5.5			2.2	
484-11	breast	(Keytruda/Taxol/Carbo)	127.6					
484-22	breast	(Keytruda)	3.3	5.2			3.1	
493-2	Lung	Keytruda/Alimta/Carbo	505.9					
503-4	breast	(Taxol/Carbo)	28.9	8.7				
503-5	breast	Adria/Cytoxan/Keytruda	7.7	1.7	3.4		1.5	
520-14	NSCLC	(Keytruda)		1.9	7.7		5.22	
524-1	Mesothelioma	Opdivo/Yervoy	7.5	6.08	5.7		9.2	
527-9	breast	(Keynote 522) Taxol/Carco/ Keytruda	2.8	4.5	1.8		8.4	8.0
527-10	breast	Adria/Cytoxan/Keytruda - Keytruda	21.1	9.5	1.5		2.5	
529-1	breast	Adria/Cytoxan/Keytruda - Keytruda	44.5	2.6				
539-6	breast	(Taxol/Carbo)	6.03	5.6				
540-6	breast	(Keytruda/Taxol/Carbo)	32.01					
543-3	Renal Cell Carcinoma	(Opdivo)	3.4	3.1			1.4	
552-1	NSCLC	(Opdivo/Alimta/Carbo)	2.6	3.9	4.6		2.8	
557-4	NSCLC	(Alimta/Carbo)		5.4	5.3		5.0	
574-14	breast	(Keytruda/AC)	55.5	2.6	1.6		3	2.1
574-21	breast	(Keytruda)		1.2	4.1		4.9	2
574-27	breast	Keytruda			3.3	18.7	4.9	
615-1	NSCLC	(Keytruda/Alimta/Carbo)	11.6	5.8	5.7	3.3	7.8	3.7
635-1	Melanoma	Keytruda	52.8					

Supplementary Table S2: Human Oligonucleotides		
**Gene**	**Forward Sequence**	**Reverse Sequence**
aldh18a1	5’-ATCTCGTCCTGACTGTCTACC-3’	5’-CCTCCTTCCCTCCTTTGAGTA-3’
arg1	5’-ACTCCACTGACAACCACAAG-3’	5’-TGGCAGATATACAGGGAGTCA-3’
b2m	5’-GGACTGGTCTTTCTATCTCTTGT-’3	5’-ACCTCCATGATGCTGCTTAC-3’
mmp9	5’-ACATCGTCATCCAGTTTGGTG-3’	5’-CGTCGAAATGGGCGTCT-3’
nos2	5’-CACCATCCTCTTTGCGTACA-3’	5’-GCAGCTCAGCCTGTACT-3’
odc1	5’-TCTACGTTCAATGGCTTCCAG-3’	5’-GCATCCTGTTCCTCTACTTCG-3’
paox	5’-GGTAGAGTGTGAGGATGGAGA-3’	5’-CACAGAGAACGTGGACAGAC-3’
sat1	5’-CAGAAGTGCCGAAAGAGCA-3’	5’-CTCATCACGAAGAAGTCCTCAA-3’
amd1	5’-ATCAGTCAGCCAGATCAAACC-3’	5’-GTATCAGGTCACGAATTCCACT-3’
smox	5’-CTCGCCGCAGACTTACTTC-3’	5’-GATTCACAACTTTGCATACCGT-3’
atp13a3	5’-TGGCAACTCATAGTACCGTAAG-3’	5’-CCATTAATAAGCACAGCATCACA-3’
gls	5’-AAGCCTGCAAAGTAAACCCT-3’	5’-GTACTGGACTTGGTATTCTTGGAG-3’
oat	5’-GTCAGACCTGATATAGTCCTCCT-3’	5’-CTGGCTTAATGGTCAGCATGA-3’
myc	5’-TCCTCGGATTCTCTGCTCTC-3’	5’-TCTTCCTCATCTTCTTGTTCCTC-3’

Supplementary Figure S1

Median Gene Expression (Polyamine Metabolism) in PBMCs

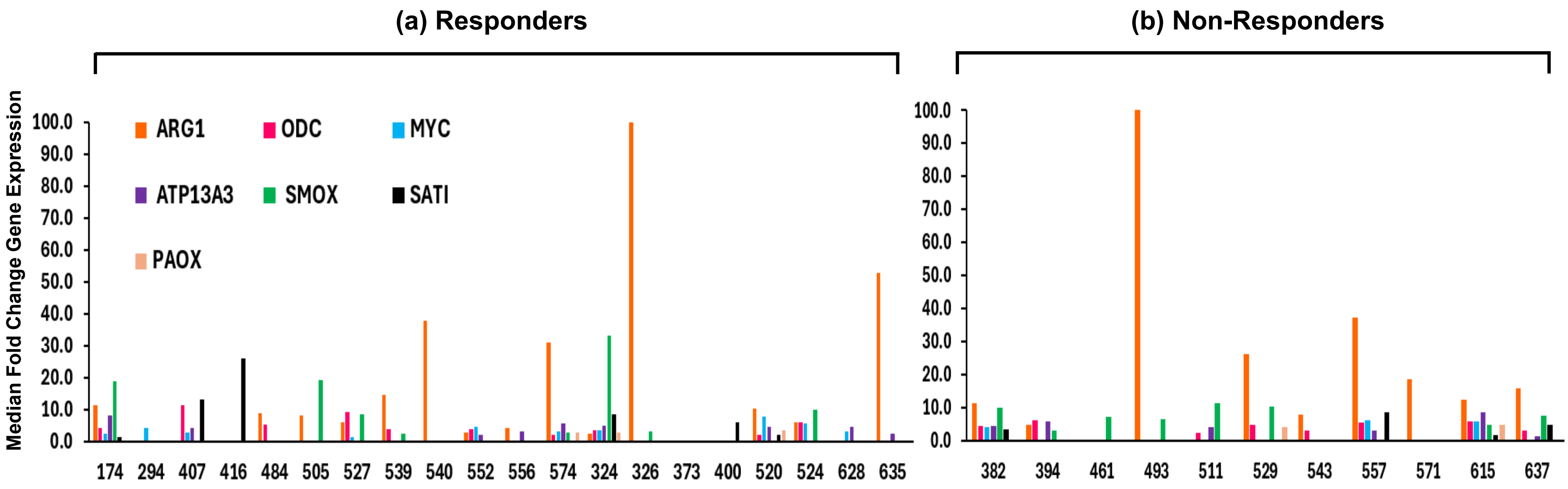

Supplementary Figure S2

Median Gene Expression (Polyamine Metabolism) in Polymorphonuclear Leukocytes

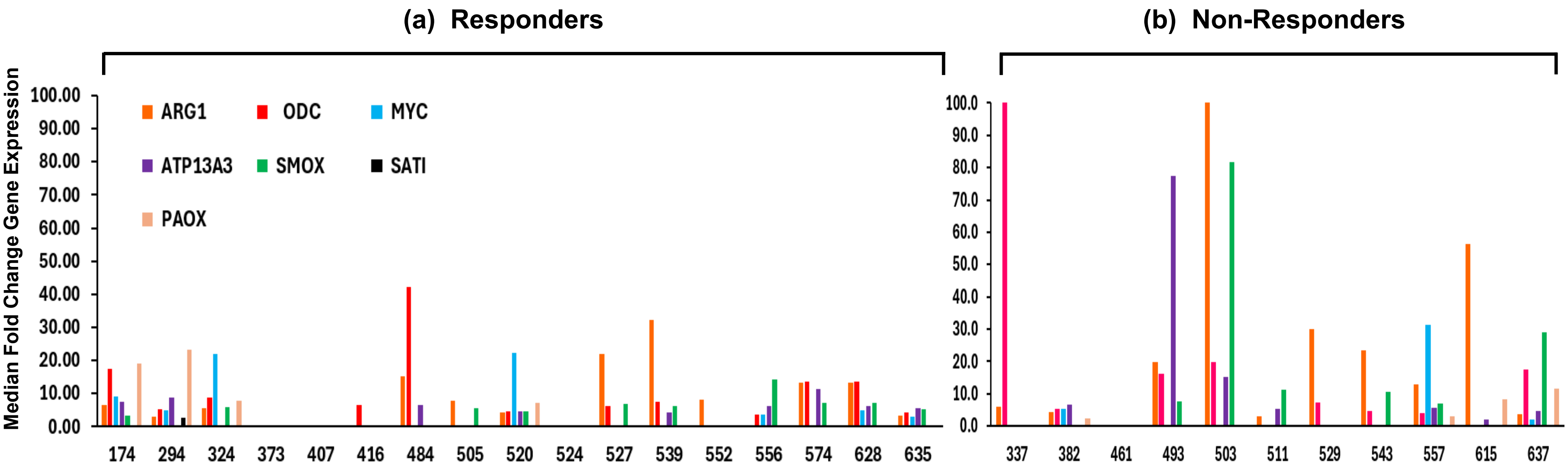

Supplementary Table S1. Correlations between Gene Expression of MYC, Arginase-1, ODC, and Glutamine Metabolism in Isolated PBMCs. qPCR was performed using RNA derived from PBMCs isolated from individual cancer patient blood samples (identified by codes names 174-79 to 635-1) and compared to that of healthy volunteers (n=5). MYC; glutamine catabolism genes include glutaminase (GLS), aldehyde dehydrogenase (ALDH18A1), and ornithine aminotransferase (OAT); polyamine metabolism-related genes include arginase-1 (ARG1) and ornithine decarboxylase (ODC). No increased gene expression in the cancer patient's PBMCs if blank.

Note highlighted correlations between:

1) increased expression of ODC and MYC (blue)

2) increased expression of ARG1 (green) and no increased expression of ODC

3) increased expression of ODC (blue) and glutamine catabolic genes (red).

Supplementary Table S2. Reverse and forward oligonucleotide primers used in qPCR reactions to quantitate human polyamine and glutamine metabolic gene expression in RNA isolated from PBMCs and PMN leukocytes isolated from peripheral blood of cancer patients and healthy volunteers.

Supplementary Figure S1. Analysis of Polyamine-Related Genes in PBMCs in Cancer Patients Responsive or Non-responsive to Immunotherapy. qPCR was performed on PBMCs isolated from peripheral blood samples (n = 135) from 29 cancer patients being treated with immune checkpoint inhibitors and from peripheral blood samples from 10 healthy volunteers. The average fold change in expression of genes in PBMCs from cancer patients compared to healthy control subjects was determined after normalizing to an internal housekeeping gene B2M. **(a)** Analysis of gene expression in PBMCs from 19 patients responding favorably to immunotherapy. **(b)** Analysis of gene expression in PBMCs from 10 patients nonresponsive to immunotherapy as defined by tumor progression or death. Polyamine metabolism-related genes include: arginase-1 (ARG1), nitric oxide synthase-2 (NOS2), ornithine decarboxylase (ODC), c-MYC, adenosylmethionine decarboxylase 1 (AMD1), polyamine transport protein ATP13A3, spermine oxidase (SMOX); spermidine/spermine N-acetyltransferase-1 (SAT1), and polyamine oxidase (PAOX).

Supplementary Figure S2. Analysis of Polyamine-Related Genes in PMNs in Cancer Patients Responsive or Non-responsive to Immunotherapy. qPCR was performed on PBMCs isolated from peripheral blood samples (n = 135) from 29 cancer patients being treated with immune checkpoint inhibitors and from peripheral blood samples from 10 healthy volunteers. The average fold change in expression of genes in PMN from cancer patients compared to healthy control subjects was determined after normalizing to an internal housekeeping gene B2M. **(a)** Analysis of gene expression in PMN from 19 patients responding favorably to immunotherapy. **(b)** Analysis of gene expression in PMN from 10 patients nonresponsive to immunotherapy as defined by tumor progression or death. Polyamine metabolism-related genes include: arginase-1 (ARG1), nitric oxide synthase-2 (NOS2), ornithine decarboxylase (ODC), c-MYC, adenosylmethionine decarboxylase 1 (AMD1), polyamine transport protein ATP13A3, spermine oxidase (SMOX); spermidine/spermine N-acetyltransferase-1 (SAT1), and polyamine oxidase (PAOX).
